# Hydrogen Absorption in Pd–Ag Systems: A TPD and Electrical Resistivity Study

**DOI:** 10.3390/ma12193160

**Published:** 2019-09-27

**Authors:** Alfonso Pozio, Zoran Jovanovic, Silvano Tosti

**Affiliations:** 1 Department of Energy Technologies, ENEA Casaccia Research Center, Via Anguillarese 301, 00123 S. Maria di Galeria (Rome), Italy; 2 Laboratory of Physics, Vinča Institute of Nuclear Sciences, University of Belgrade, P.O. Box 522, 11001 Belgrade, Serbia; 3 Center of Excelence for Hydrogen and Renewable Energy (CONVINCE), Vinča Institute of Nuclear Sciences, University of Belgrade, P.O. Box 522, 11001 Belgrade, Serbia; 4 Division of Fusion Physic, ENEA Frascati Research Center, Via E. Fermi 45, 00044 Frascati (RM), Italy

**Keywords:** Pd–Ag alloy, hydrogen, temperature-programmed desorption, electrical resistivity

## Abstract

Hydrogen retention in Pd–Ag (silver 21 wt. %) thin foil has been tested by means of temperature-programmed desorption (TPD) in the temperature range 25–200 °C and compared to the resistivity measurements for the purpose of explaining the characteristic S-shaped resistivity curve and its minimum observed in the same temperature range. The TPD results indicated that the highest uptake of hydrogen was between 65 °C and 105 °C, with a maximum at ~85 °C. Furthermore, in all examined cases, the hydrogen desorption peak was between 140 °C and 180 °C. The resistivity measurements in argon, hydrogen, and vacuum allowed us to examine the influence of hydrogen on the resistivity of a Pd–Ag alloy. The results showed evidence of two kinds of hydrides: (1) a weak absorption at low temperature (T < 70 °C) with the hydrogen present mainly in tetrahedral sites, and (2) a strong absorption up to 150 °C with the hydrogen present mainly in octahedral sites. The behaviour of the electrical resistivity and the minimum between 90 °C and 110 °C can be explained by the two kinds of hydrogen uploaded into the metal lattice.

## 1. Introduction

Palladium-based membranes are important for technological processes such as the separation and purification of hydrogen [1,2]. In order to avoid hydrogen embrittlement, an alloy of Pd and Ag is generally made [3,4]. Indeed, a hydrogenated Pd-membrane becomes brittle after certain cycles of α ↔ β hydride transformation due to its lattice expansion. Conversely, in Pd–Ag alloys, the lattice has already been expanded by the silver atoms, so it is less influenced by hydrogen uploading and, consequently, less brittle than the pure Pd [1]. The alloying with Ag also improves the mechanical properties and the hydrogen permeability [5]. In fact, both the tensile strength and the permeability show a maximum for a silver content in the range 20–40 wt.%, a content of Ag in the commercial Pd alloys used for membrane applications. It is also noteworthy that the electrical resistivity of non hydrogenated Pd–Ag alloys reaches a maximum silver content in the range 20–40 wt. % [6]. Among different design configurations, the direct ohmic heating of Pd–Ag permeator tubes has been studied. Such an arrangement permitted the reduction of power consumption with respect to the traditional membrane module heating systems and shortened temperature ramping [5]. These applications evidenced the need to study the effects of the metal/hydrogen interactions on the electrical resistivity of the Pd alloy in more detail. The resistivity of a Pd_1−x_–Ag_x_ alloy shows a typical S-shape curve as a function of x (wt. %), appearing at x = 10 and disappearing completely at x = 50 [7,8,9]. This range of the S-shape curve is also influenced by the hydrogen uploading, and in presence of H, the x is reduced to the range of 19–29. Generally, several works agreed that the S-shaped trend vs. the H/M ratio for x in this range could be affected by the hydrogen uploading into the metal lattice, involving the presence of different hydride phases: an α-phase for low H/M, an α + β two-phase co-existence region for higher H/M, and then a β phase [7,8,9]. The exact phase limits should depend on the silver content, temperature, and pressure, and their increase reduces the two-phase region [3,10]. Nevertheless, the S-shape behavior of Pd–Ag resistivity remained unclear, as well as the minimum at a higher H/M ratio with its subsequent increase [11].

The low-temperature inversion (80–150 °C) in the electrical resistivity curve of Pd–Ag was observed and explained with consideration of the two different hydride forms present in its lattice [11,12,13]. A low-temperature hydride form (tetrahedral positions occupied by H) is expected to have a higher resistivity than a high-temperature one (octahedral), so a minimum of the resistivity for hydrogenated Pd–Ag appears in this temperature range [12,13].

Leary et al. have used a temperature-programmed desorption (TPD) method to evidence the penetration of hydrogen into subsurface absorption sites of silica-supported palladium [14]. The authors claimed that some of the hydrogen that is initially adsorbed on the surface at room temperature, penetrates into subsurface absorption sites during heating of the palladium crystallites. When the surface becomes depleted of hydrogen by desorption, the subsurface hydrogen moves back to the surface and desorbs in a high-temperature peak.

Baumberger et al. derived a surface structural model for the description of hydrogen chemisorption phases formed on Pd (110) [15]. On the basis of structural arguments, the authors suggested that the subsurface sites, corresponding to the octahedral interstitials between the topmost and second atomic layers, are populated by thermal activation.

We can suppose that the existence of different kinds of hydride forms is more pronounced in the Pd–Ag lattice where the difference between the hydrogen position in the lattice is more evident, as shown by resistivity data [11]. In this work, TPD and resistivity measurements were coupled in order to understand the hydrogen absorption process, its role in electrical resistivity, and its correlation to the existence of different hydride forms in hydrogenated Pd–Ag alloys in the temperature range 25–200 °C.

## 2. Experimental Methods

### 2.1. The Activation of Pd–Ag Foils

**Figure 1:**
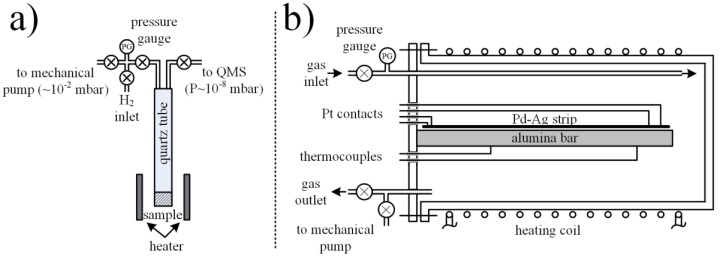
The schematic representation of (**a**) temperature-programmed desorption and (**b**) resistivity experimental setups.

The foils of Pd_0.79_–Ag_0.21_ and Pd_0.30_–Ag_0.70_ were cleaned in acetone, ethanol, and water, respectively, for 15 min in ultrasound and dried in an oven at 130 °C for 30 min. Next, the foils were activated by heating in Ar at 250 °C for 1 h, followed by a 1 h treatment in H_2_ at 350 °C and cooled in Ar at 250 °C for 1 h, before being allowed to cool to room temperature. The gas flow was 30 mL min^−1^ in all the cases. Then, the foils were instantly placed on the bottom of the quartz tube (Figure 1a) for absorption/desorption tests.

### 2.2. Absorption/Desorption Tests and Resistivity Measurements

The quartz tube (Figure 1a) was positioned in the center of the heating zone and calibration was performed to provide accurate temperature measurement in the foil vicinity (Figure 1a). Prior to hydrogen uploading experiments, the system was vacuumed for 15 min by a roughing pump, purged with H_2_ at room temperature (~30 s total exposure), and vacuumed again. Next, the system was filled with hydrogen at ~10^5^ Pa at room temperature and absorption experiments were performed with T_abs_ from 25 to 200 °C by allowing a sufficient amount of time (~90 min, on average) for pressure equilibration due to hydrogen uploading into the foil. After pressure equilibration, the hydrogen was vacuumed by a roughing pump (2–3 min) and desorption measurements were started. In this process, the temperature was increased from the T_abs_, at a rate of 10 °C/min, up to 450 °C, while the desorbed hydrogen was monitored by a quadruple mass spectrometer (QMS, XT300, Extorr Inc. New Kensington, PA, USA)) which was mounted on a homemade TPD set-up.

The resistivity measurements were performed in four-electrode AC impedance mode using a Solartron 1260 frequency response analyzer by Schlumberger. The spectra were recorded continuously at 10 Hz with 10 points per decade and a maximum perturbation amplitude of 0.5 mA.

The same experimental setup as that described in a previous work was used [11]. In brief, a Pd–Ag 21 wt. % thin foil was cut into a strip of length 14.10 cm and width 0.44 cm that was positioned in a gas-tight stainless steel module. Four parallel platinum contacts were welded onto the ends of the Pd–Ag strip. The length (*l*) of the Pd–Ag sample between the measuring voltage probes was 13.75 cm. The impedance of the foil was measured in the tangential direction (in-plane) and the resistivity was calculated using the equation:

(1) $$\rho =\frac{R_{\Omega }\times A}{l}$$

where *ρ*, *l*, *R*_Ω_, and *A* denote the resistivity, the distance between the probe, the measured resistance, and the cross-sectional area of the Pd–Ag sample, respectively. The term *A* was obtained from the product of the Pd–Ag thickness (*r*) 51 µm and its width (*h*) 0.44 cm.

The closed stainless steel vessel container provided with a gas inlet and outlet was equipped with an electrical heating system and two control thermocouples (Figure 1b). This vessel was thermally insulated and small enough to ensure a uniform temperature in the entire volume.

The resistivity and temperature were continuously measured during the entire experiment, which was performed in stages. In stage 1, the experiment started with argon flow (15 mL min^−1^) at room temperature, and we waited for a stable signal. Then, in stage 2, the temperature was increased, in argon, to an absorption temperature (T_abs_). In stage 3, at T_abs_, the vessel was purged with H_2_ (15 mL min^−1^), and we waited again for signal stabilization. In stage 4, the vessel was closed and the hydrogen was vacuumed by a mechanical pump, and we waited for a stable signal. In stage 5, the temperature was increased from the T_abs_, at a rate of 5 °C min^−1^, up to the final temperature (about 450 °C), while always monitoring the resistivity. At the end of the heating ramp, in stage 6, a flow of argon was sent into the vessel, cooling the system until room temperature was reached. This procedure was applied for each T_abs_ temperature.

## 3. Results and Discussion

### 3.1. Resistivity in A Pd–Ag–H_n_ (n = H/Me) System in Argon and Hydrogen Atmosphere

**Figure 2:**
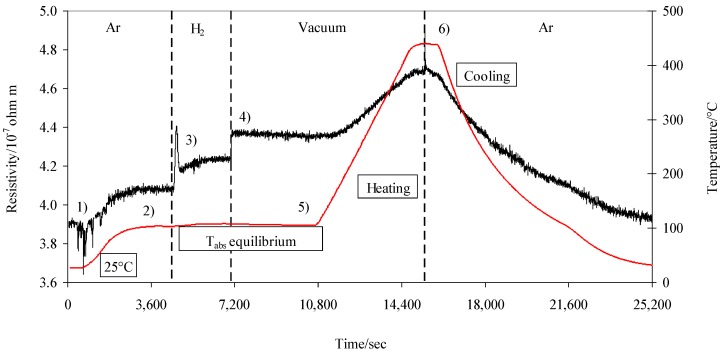
Resistivity and temperature during a typical impedance measurement.

Figure 2 shows the resistivity and temperature during a typical impedance measurement following the above-mentioned procedure.

**Figure 3:**
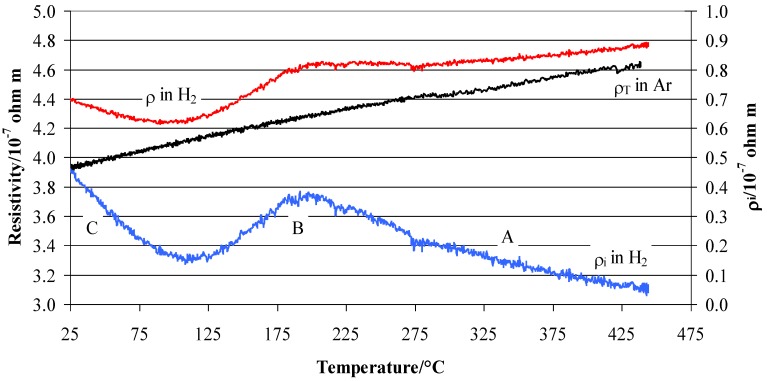
Total resistivity (ρ), contribution due to the scattering measured in pure hydrogen (ρ_i_), and contribution of ρ_T_ measured in argon of Pd–Ag 21 wt. % thin foil. For all temperatures, the measurements were performed at 100 kPa of hydrogen.

The total resistivity of Pd_79_–Ag_21_–H_n_ (n = H/Me) can be defined as the sum of two terms [11,15]:

(2) $$\rho =\rho_{T}+\rho_{i}$$

where ρ_T_ is the resistivity due only to the thermal motion of the hydrogen-free Pd–Ag lattice. This is the typical behaviour of a nonhydrogenated metal that can described by the simplified equation:

(3) $$\rho_{T}=\rho {}^{\circ}\;[1+\alpha \;(T-T{}^{\circ})]$$

where T is the temperature, T° is a reference temperature (usually room temperature), ρ° is the resistivity at T°, and α is the change in resistivity per unit temperature and is called the thermal coefficient. ρ° and α can be obtained by measuring the resistivity in flowing argon as a function of temperature (Figure 3).

The ρ_i_ in Equation (2) is the resistivity due to scattering of the free electrons on the metal’s and hydrogen atoms. This term clearly depends on the H concentration in the sample, which, at constant H_2_ gas pressure, changes with temperature due to a temperature-dependent H solubility. From Equations (2) and (3), we can calculate this contribution simply by subtracting the resistivity measured in argon from that in pure hydrogen (Figure 3):

(4) $$\rho_{i}=\rho -\rho {}^{\circ}\;[1+\alpha \;(T-T{}^{\circ})]$$

The ρ_i_ contribution is strictly dependent on the hydrogen trapped in the lattice in relation to the total amount of silver. Particularly for Ag 21 wt. %, the hydrogen atoms can occupy octahedral and tetrahedral positions [13]. Before the *d*-band is filled, all hydrogen atoms occupy the octahedral positions at high temperatures and the tetrahedral positions at low temperatures. However, at higher hydrogen concentrations, the tetrahedral interstitial positions begin to be occupied too. We obtain the relation:

(5) $$n=n^{tet}+n^{oct}$$

According to this model [13], the s-band density of states is assumed to be constant in the diamagnetic state, i.e., only when x + n ≥ 0.6, does the s-band starts to accept electrons from hydrogen [16,17]. This means that, for Ag 21%, if n ≥ 0.39, the amount of hydrogen in a tetrahedral position should increase, and for n ≤ 0.39, the amount of *n*^tet^ should be equal to zero.

The ρ_i_ contribution due to the hydrogen inside the lattice can change or disappear totally after a vacuum/desorption process, depending on the temperature. So, at every T_abs_, a resistivity in flowing hydrogen (ρ_i_) and in a vacuum (ρ_i_′) can be measured. In addition, the vacuum process always reduces n ≤ 0.39. In this case, the exact position of hydrogen, tetrahedral or octahedral, will depend on the T_abs_. So the parameter ρ_i_′ can be considered to be dependent on the hydrogen trapped in the lattice position at T_abs_ after the vacuum process.

### 3.2. Thermal Desorption of Hydrogen from A Pd–Ag Alloy.

The experimental tests have been focused on studying the absorption/desorption behavior of hydrogen in Pd–Ag alloys. In a preliminary TPD test, samples with two different Ag loadings (in wt.%), Pd_0.79_–Ag_0.21_ and a Pd_0.30_–Ag_0.70_, were compared, evidencing that the alloy composition plays a determining role in hydrogen absorption/desorption behavior. In particular, Pd_0.30_–Ag_0.70_ showed no H_2_ desorption, while Pd_0.79_–Ag_0.21_ showed desorption up to T_abs_ = 160 °C. As explained in a previous article [11], the 40 wt. % of silver in the alloy irreversibly modified the behavior of the alloy so that it cannot absorb any kind of hydrogen due to its electronic structure. The TPD effectively evidenced this effect, showing no hydrogen absorption/desorption for 70 wt.% of Ag.

**Figure 4:**
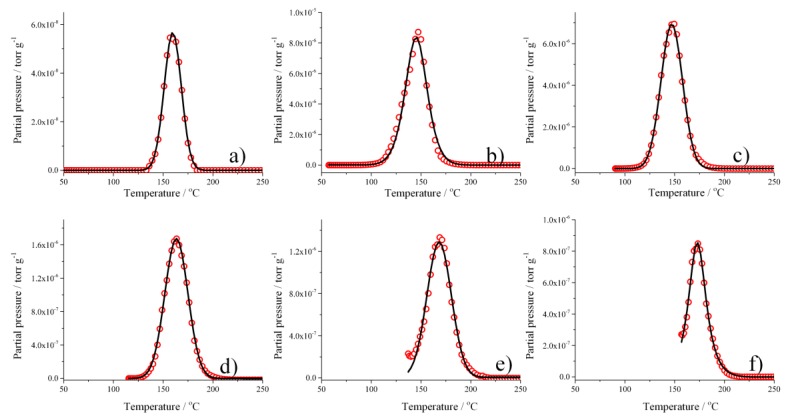
Thermal desorption of H_2_ from Pd_79_–Ag_21_ foil after absorption at: (**a**) 25 °C; (**b**) 60 °C; (**c**) 100 °C; (**d**) 120 °C; (**e**) 140 °C; and (**f**) 160 °C.

The TPD was used to analyze alloys with 21 wt. % Ag in more detail. The absorption of H_2_ was performed at T_abs_ 25–160 °C, and the results are shown in Figure 4. As can be seen, detectable hydrogen desorption can be already observed at ~100 °C, with maxima between 140 and 180 °C, thus representing a region of thermal stability of the hydrogen–metal bond.

**Figure 5:**
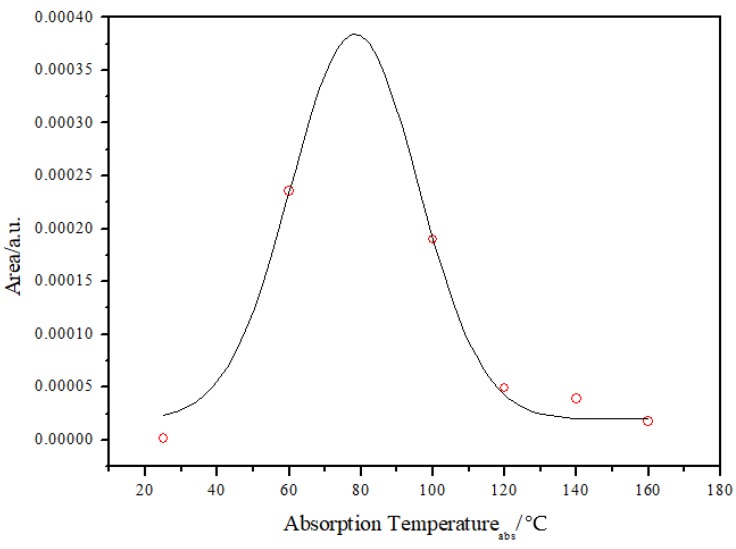
The total area of H_2_ absorbed in Pd_79_–Ag_21_ foil for different absorption temperatures.

The area under the TPD curves can be used for a relative comparison of the amount of hydrogen absorbed by the Pd–Ag lattice at a well-defined absorption temperature (T_abs_), remaining after the vacuum process, and then recovered by desorption (Figure 5). It can be concluded that the highest uptake of hydrogen was achieved between T_abs_ ~63 °C and 105 °C, with a maximum at ~85 °C.

In the following discussion about the behaviour of the electrical resistivity of hydrogenated Pd–Ag, we will try to identify the origin of hydrogen from the perspective of its bonding in the Pd–Ag lattice.

### 3.3. Resistivity of Pd_0.79_–Ag_0.21_ in Hydrogen and a Vacuum and Its Link to Hydride Forms

**Figure 6:**
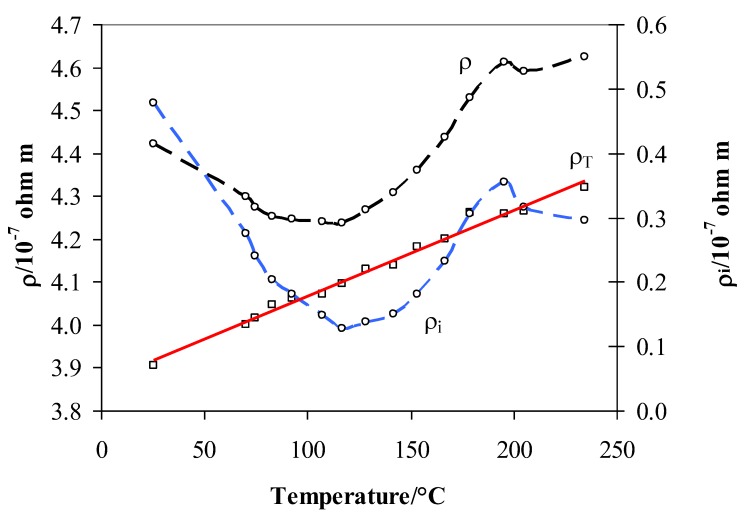
Total resistivity ρ and its contribution due to the scattering ρ_i_ of Pd_79_–Ag_21_ foil measured in 100 kPa hydrogen at different temperatures.

**Figure 7:**
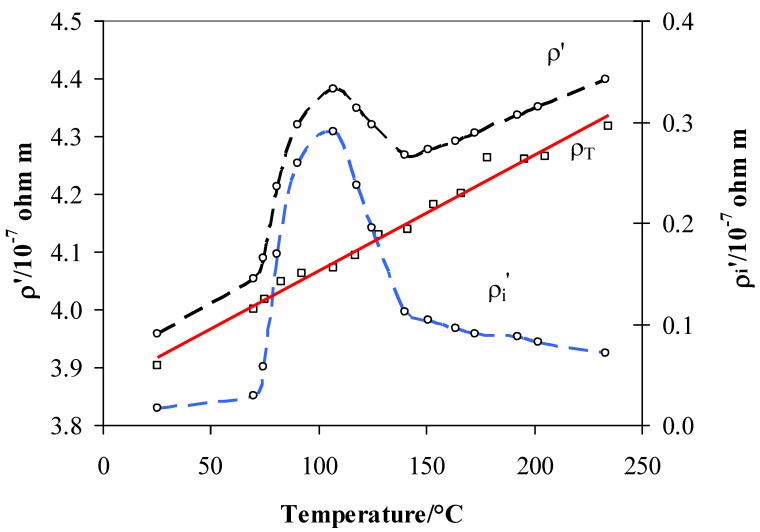
Total resistivity ρ′ and its contribution due to the scattering ρ_i_′ of Pd_79_–Ag_21_ foil measured after the vacuum step at different temperatures.

Figure 6 shows the temperature evolution of the three terms of Equation (2) in the range 25–235 °C. As in Figure 3, we can distinguish a minimum of ρ_i_ at about 110 °C and the maximum at 200 °C. Similarly, Figure 7 shows the total resistivity ρ’ and the intrinsic resistivity ρ_i_′ after the vacuum step at different T_abs_. So, we attempt to establish a relationship between the residual intrinsic resistivity ρ_i_′ after the vacuum process and the hydrogen presence inside the lattice as observed by the TPD measurements.

It can be noticed that the maximum ρ_i_′ appeared in the same range of absorption temperatures (90–110 °C) as in the case of the TPD experiment (Figure 5). This evidenced that the amount of hydrogen extracted by the vacuum was correlated to the absorption temperature. This hydrogen, as in the TPD measurement (Figure 4), could be extracted completely only by increasing the temperature to over 200 °C.

To explain the trend of resistivity obtained in hydrogen after the vacuum process, when the excess H_2_ was pumped out, we must first consider how the absorption temperature and the amount of hydrogen determined the position of the hydrogen inside the lattice and its role.

**Figure 8:**
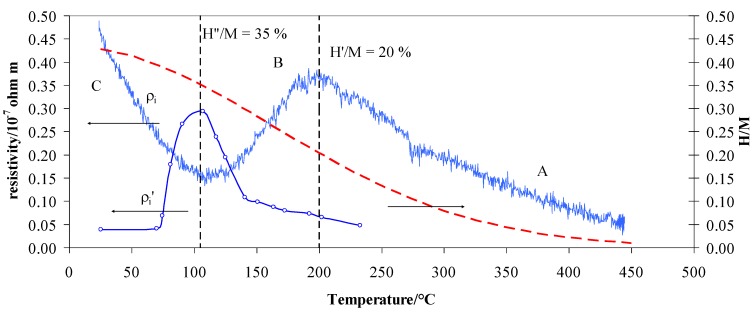
The resistivity contribution due to the scattering of ρ_i_ measured in pure hydrogen and in a vacuum (ρ_i_’) with the H/M vs. temperature in pure hydrogen at 100 kPa.

According to the literature, we can summarize the situation in a hydrogen atmosphere as in [13]. So, the vacuum process after absorption at different temperature acted on different kinds of hydrogen. From 25 to 70 °C, the vacuum process extracted practically all of the hydrogen present in the tetrahedral sites so that the resistivity ρ_i_′ was about zero. By increasing the temperature to above 70 °C, the equilibrium shifted from the tetrahedral to the octahedral sites, and the residual resistivity ρ_i_′ increased. The growth of ρ_i_′ with the absorption of temperature is explained by an increase of hydrogen inside the lattice due to the shift in the tetrahedral ↔ octahedral equilibrium. The vacuum process seemed to extract the hydrogen completely from the tetrahedral sites (25–70 °C) but only partially from the octahedral sites (T > 70 °C). Now, we can observe that this amount of hydrogen, which still remained inside the lattice in octahedral sites, filled the *d*-band since the H/M ratio was lower than 0.2 (Table 1). Consequently, this hydrogen produced an increase of resistivity with respect to that in pure argon, therefore, we observed a maximum of resistivity ρ_i_’ at ~110 °C. Thus, the maximum can be interpreted as a complete shift of the tetrahedral ↔ octahedral equilibrium. Above this temperature, the vacuum, also assisted by desorption process, would extract hydrogen only from the octahedral sites, so a successive increase of temperature would reduce the H/M ratio. As in the previous case, hydrogen that still remained inside the lattice would occupy the *d*-band, but as the temperature increased, this amount would be lowered (H/M reduces with temperature), so the residual resistivity ρ_i_′ tended to decrease. While ρ_i_′ was correlated to the amount of absorbed hydrogen, as shown by the relative trend in Figure 5, the TPD results also revealed the range of temperature stability for hydrogen in a Pd–Ag alloy, which helps us to understand the trend of ρ_i_ in Figure 8. As can be seen in Figure 4, desorption of hydrogen occurred between 100 and 200 °C, but in the case of higher T_abs_, the desorption process was already active (Figure 4e,f). Thus, in the case of measurements in flowing hydrogen, resistivity ρ_i_ can be understood as a net result of a complex absorption/desorption process whose constants, depending on the temperature, determine the H/M content in the alloy.

The Pd–Ag resistivity measured in hydrogen (Figure 3) can be now overlapped by that obtained after the vacuum/desorption process at different absorption temperatures. Here, H/M was extrapolated by the literature data on the same kind of alloy [11,18,19,20,21,22]. By moving from right to left of the graph in Figure 8, i.e., following the temperature decrease starting from 450 °C, it is observed that: (1) in region A below 450 °C, the resistivity ρ_i_ increases until a maximum was reached at ~200 °C, (2) in region B, a decrease is observed until a minimum in resistivity is reached at ~110 °C, and (3) in region C, there is a continuous increase of resistivity. Such a trend can be explained due to the fact that the hydrogen uploaded into different lattice positions affects the resistivity in a different way. The hydrogen uploaded at high temperatures (200–450 °C) increases the resistivity, while that uploaded below 200 °C decreases it. In a previous article [11], we hypothesized that introducing H into a Pd_79_–Ag_21_ lattice initially decreases the number of metal valence electrons (N), thus increasing the resistivity ρ_i_. This effect occurs until the electrons fill the valence band (saturation effect) at a well-defined loading of hydrogen H’ until the sum of H’/M + 0.21(Ag) reaches about 40% [16,17,23]. Effectively, we observed a maximum of (H′/M) = 0.20 at about 200 °C. Before the *d*-band is filled, all these hydrogen atoms occupied interstitial positions of one kind only, in this case at high temperatures, hydrogen occupied the octahedral positions.

Further uptake of hydrogen involves an increase of N to decrease resistivity ρ_i_ until we reach a higher H″/M ratio. After the *d*-band is filled, hydrogen continues to occupy octahedral interstitial positions, but as the H/M increases tetrahedral positions can also be occupied. The theoretical limit of H/M for the octahedral position should be reached for n ≤ 0.39. In contrast to the model [13], the resistivity ρ_i_ in Figure 8 decreases until H″/M is 0.35 at 105 °C. This can be explained by considering that the limit between octahedral and tetrahedral positions is not as well defined. In addition, it should be considered that the H/M at a low temperature is affected by measurement uncertainty. However, we can observe that above a threshold value (H″/M), the hydrogen is also in the tetrahedral position.

From the TPD and resistivity results in the range 25–200 °C, we can clearly see the transition between the two different hydrides. The vacuum process reduces the amount of hydrogen well under the filling of the *d*-band, so the hydrogen atoms, if present, can occupy interstitial positions of one kind only. Measurements show that the hydrogen amount still inside the alloy increases as we shift toward higher absorption temperatures. The maximum of this hydrogen is reached at an absorption temperature corresponding to that which we observed at the minimum resistivity. As predicted from the model [13], this point should represent the limit of existence for the *n*^tet^. We can conclude that this hydrogen is located in a well-defined octahedral site when thermally activated, and occupies a well-defined range of temperatures.

Above this H″/M ratio at lower temperatures, the tetrahedral interstitial positions also begin to be extensively occupied. The scattering effect is dominant with respect to the numbers of conduction electrons per unit volume—the high concentration of H atoms, always occupying more tetrahedral sites, act mainly as a point defect, thus decreasing the electrons mean free paths and sharply increasing the resistivity.

The experiment on TPD and resistivity in vacuum conditions demonstrated the existence of different kinds of hydride that can be activated by means of temperature by starting from about 70 °C. This activation effect was already evidenced in previous electrochemical measurements. Pozio et al. [24] used a similar Pd–Ag cathode in an experimental alkaline electrolyser and observed characteristic changes in the slope of a permeating flux of H_2_ for different temperatures in 1 M KOH. Their results showed that the permeating flux logarithm is a linear function of 1/T, with a slope change at about 70 °C. Particularly, an activation energy of 34.7 kJ mol^−1^ was calculated between 50 and 70 °C, and 4.0 kJ mol^−1^ between 70 and 80 °C. This change of slope corresponds to the change of resistivity ρ_i_′ evidenced in Figure 7 and Figure 8 at the same temperature. We can presume that above 70 °C, the hydrogen undergoes thermal activation, and it can occupy and move across octahedral lattice sites, thus changing the physical parameters, such as resistivity, absorption, and permeability.

## 4. Conclusions

An experimental study based on TPD and electrical resistivity measurements was utilized to explain the characteristic S-shaped curve of electrical resistivity of a Pd–Ag foil in the temperature range 25–200 °C. Measurements showed that two different hydrides exist with their conversions starting at ~70 °C. At low temperatures, a weak absorption of hydrogen is present, mainly in tetrahedral sites. At higher temperatures, a stronger absorption occurs until 150 °C, with the hydrogen present mainly in octahedral sites. The behaviour of the electrical resistivity and the minimum between 90 and 110 °C was discussed from the perspective of the effects these hydride forms have on host metal lattices. This was enabled by the new approach that combined TPD and resistivity measurements in a vacuum, thus providing a novel insight into the interactions and temperature stability of hydrogen in a Pd–Ag alloy. Thanks to this, we were able to establish a relationship between the residual intrinsic resistivity after the vacuum/desorption process, hydrogen presence inside the lattice, and its role in the overall evolution of resistivity obtained in a hydrogen atmosphere.

## Figures and Tables

**Table 1:** The hydrogen position in the Pd_79_–Ag_21_ lattice vs. H/M, temperature, related role, and resistivity trends.

Temperature/°C	H/M	H Position	Resistivity	Role
>200	0–0.20	Octahedral	Increase	*d*-band filling
200–110	0.20–0.39	Octahedral	Decrease	Number of valence e^−^ increases
<110	>0.39	Octahedral ↔ Tetrahedral	Increase	Scattering effect
